# Highly Stable and Photoluminescent CsPbBr_3_/Cs_4_PbBr_6_ Composites for White-Light-Emitting Diodes and Visible Light Communication

**DOI:** 10.3390/nano13020355

**Published:** 2023-01-15

**Authors:** Longshi Rao, Bin Sun, Yang Liu, Guisheng Zhong, Mingfu Wen, Jiayang Zhang, Ting Fu, Shuangxi Wang, Fengtao Wang, Xiaodong Niu

**Affiliations:** 1 Department of Mechanical Engineering, College of Engineering, Shantou University, Shantou 515063, China; 2 Intelligent Manufacturing Key Laboratory of Ministry of Education, Shantou University, Shantou 515063, China; 3 Hubei Key Laboratory of Mechanical Transmission and Manufacturing Engineering, Wuhan University of Science and Technology, Wuhan 430081, China

**Keywords:** ultrasonication, liquid paraffin, CsPbBr_3_/Cs_4_PbBr_6_, white-light-emitting diodes, visible light communication

## Abstract

Inorganic lead halide perovskite is one of the most excellent fluorescent materials, and it plays an essential role in high-definition display and visible light communication (VLC). Its photochromic properties and stability determine the final performance of light-emitting devices. However, efficiently synthesizing perovskite with high quality and stability remains a significant challenge. Here, we develop a facile and environmentally friendly method for preparing high-stability and strong-emission CsPbBr_3_/Cs_4_PbBr_6_ composites using ultrasonication and liquid paraffin. Tuning the contents of liquid paraffin, bright-emission CsPbBr_3_/Cs_4_PbBr_6_ composite powders with a maximum PLQY of 74% were achieved. Thanks to the protection of the Cs_4_PbBr_6_ matrix and liquid paraffin, the photostability, thermostability, and polar solvent stability of CsPbBr_3_/Cs_4_PbBr_6_-LP are significantly improved compared to CsPbBr_3_ quantum dots and CsPbBr_3_/Cs_4_PbBr_6_ composites that were prepared without liquid paraffin. Moreover, the fabricated CsPbBr_3_/Cs_4_PbBr_6_-LP-based WLEDs show excellent luminescent performance with a power efficiency of 129.5 lm/W and a wide color gamut, with 121% of the NTSC and 94% of the Rec. 2020, demonstrating a promising candidate for displays. In addition, the CsPbBr_3_/Cs_4_PbBr_6_-LP-based WLEDs were also demonstrated in a VLC system. The results suggested the great potential of these high-performance WLEDs as an excitation light source to achieve VLC.

## 1. Introduction

With the intensification of competition since 2015, the large-scale application of semiconductor light-emitting diode (LED) devices in low-value fields such as lighting has seen industry profits drop below 10%. The key to solving this crisis is to develop the application of LED in high-value fields, such as high-definition displays and visible light communication [1,2]. Their technical requirements are mainly focused on high quality and reliability. The high quality of the devices depends on the photochromic performance of luminescent materials such as quantum dots and phosphors [3,4]. In contrast, the high reliability of devices is mainly affected by the stability of fluorescent materials, such as light, heat, and solvent stability [5].

Inorganic cesium lead halide perovskite (CsPbX_3_, where X = Cl, Br, or I), possessing high photoluminescence quantum yield (PLQY) [6,7], narrow photoluminescence (PL) full width at half maximum (FWHM) [8,9], and a widely tunable bandgap [10], has become one of the most appealing luminescent materials for optoelectronic devices. Despite all these advantages, owing to its low formation energy, labile surface, and metastable structure, CsPbX_3_ is extremely sensitive to moisture, heat, and light, which leads to poor stability and durability [11,12]. Besides that, the PLQY of CsPbX_3_ rapidly declines as it transforms from liquid to solid [13]. These defects seriously hamper perovskite particles’ practical applications in optoelectronic devices. Therefore, exploring a novel strategy to improve perovskite stability that can withstand the impact of heat, light, and polar solvents and keep high PLQY in the solid state is imperative.

Until now, many efforts have been made to solve the above issues. They can be classified into three strategies: (i) encapsulating the CsPbX_3_ in inorganic oxides [14,15,16], mesoporous materials [17,18,19], or hydrophobic polymers [20,21]; (ii) modifying the CsPbX_3_ surface with potent binding ligands [22,23,24]; and (iii) constructing different perovskite composite structures [25,26,27]. Among these strategies, it is found that building different perovskite composites, such as CsPbBr_3_/Cs_4_PbBr_6_ and CsPbBr_3_/Cs_2_PbBr_5_, is a very effective way to stabilize perovskite particles and maintain their high PLQY in the solid state. Researchers such as Bao et al. achieved high-stability CsPbBr_3_@Cs_4_PbBr_6_ nanocrystals (NCs) using the hot injection method, and the as-synthesized CsPbBr_3_@Cs_4_PbBr_6_ NCs were used to fabricate quantum dot LED devices with the highest current efficiency of 4.89 cd/A [28]. Moreover, He et al. fabricated a white LED (WLED) device using CsPbBr_3_/Cs_4_PbBr_6_ with a high PLQY of 60.4%. The device exhibits good stability, and the color gamut is as wide as 126% of NTSC, reaching 94% of Rec. 2020 [29]. These extraordinary achievements motivate us to design such composite materials for high-quality and reliable optoelectronic devices.

Our previous research found that mechanochemical synthesis is an effective method to achieve high-stability CsPbBr_3_/Cs_4_PbBr_6_ composites and high-quality LED devices [30,31]. For example, our group developed a high-power ultrasonication to synthesize solid CsPbBr_3_/Cs_4_PbBr_6_ composites [31]. Based on this composite material, the photostability, thermal stability, and polar solvent stability of CsPbBr_3_/Cs_4_PbBr_6_ are improved compared with CsPbBr_3_. When we further investigated the remarkable performance of this composite structure, we found that the luminescent center of CsPbBr_3_/Cs_4_PbBr_6_ is derived from CsPbBr_3_, and Cs_4_PbBr_6_ plays a role in passivating CsPbBr_3_ crystals and inhibiting their agglomeration and regrowth. Since CsPbBr_3_ is distributed inside and on the surface of Cs_4_PbBr_6_, when filtered and dried, CsPbBr_3_ distributed on the surface of Cs_4_PbBr_6_ is easily oxidatively decomposed due to a lack of ligand protection, thereby reducing the performance of CsPbBr_3_/Cs_4_PbBr_6_ composites.

This work addresses the above issue by introducing ultrasonication and a polar-free solvent, liquid paraffin, to synthesize CsPbBr_3_/Cs_4_PbBr_6_ composites. Replacing part of the DMSO with liquid paraffin not only makes the preparation process more environmentally friendly but also improves the stability of CsPbBr_3_/Cs_4_PbBr_6_ composites and the reliability of optoelectronic devices. A bright green emissive CsPbBr_3_/Cs_4_PbBr_6_ composite solid with a maximum PLQY of 74% was obtained by tuning the contents of liquid paraffin. Compared with CsPbBr_3_ and CsPbBr_3_/Cs_4_PbBr_6_ composites prepared without liquid paraffin, CsPbBr_3_/Cs_4_PbBr_6_-LP prepared using liquid paraffin has noticeable improvements in UV light stability, thermal stability, and polar solvent stability. Moreover, the as-prepared CsPbBr_3_/Cs_4_PbBr_6_-LP was used as a phosphor to fabricate high-performance WLEDs. The fabricated CsPbBr_3_/Cs_4_PbBr_6_-LP WLED show high quality and good thermal reliability, which makes them promising for display applications. Owing to the short fluorescence lifetime of perovskites, the visible light communication (VLC) potential of the CsPbBr_3_/Cs_4_PbBr_6_-LP is also investigated. These results show that the CsPbBr_3_/Cs_4_PbBr_6_-LP presents an extraordinary performance in high-definition display and VLC applications.

## 2. Materials and Methods

### 2.1. Chemicals and Materials

We purchased cesium bromide (CsBr, 99.9%), lead bromide (PbBr_2_, 99.99%), liquid paraffin (LP, AR), dimethyl sulfoxide (DMSO, 99.9%), oleic acid (OA, 90%), oleylamine (OAm, 90%), n-hexane (99.5%), and ethanol (75%) from Shanghai Aladdin Biochemical Technology Co. (Shanghai, China). Polydimethylsiloxane (PDMS) was obtained from Dow Corning Co. (Midland, MI, USA), and the red phosphor (Ba, Ca, Sr)_3_SiO_5_:Eu was received from Nichia Co. Shenzhen PANIKE Instrument Equipment Co. (Shenzhen, China), LTD supplied deionized water (H_2_O, 18.2 MΩ). All scientific reagents and materials used in this work are unpurified.

### 2.2. Synthesis of CsPbBr_3_/Cs_4_PbBr_6_ Microcrystals

**Figure 1:**
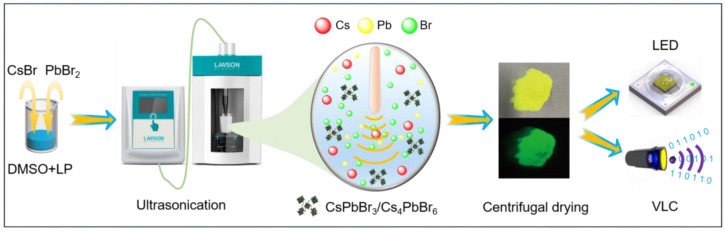
The procedures for the preparation of CsPbBr_3_/Cs_4_PbBr_6_ microcrystal using an ultrasonic processor at room temperature and their application in white-light-emitting diodes and visible light communication.

The highly green-emitting CsPbBr_3_/Cs_4_PbBr_6_ microcrystals were prepared according to our previous method by the facile ultrasonic method shown in Figure 1. First, 14.58 mmol CsBr, 2.43 mmol PbBr_2_, 0.8 mL liquid paraffin (LP), and 3.2 mL DMSO were mixed in a glass bottle. Then, the hybrid solution was sonicated for 30 min at 90 W of ultrasound power using an ultrasonic processor. Subsequently, unreacted precursors were removed by centrifugation at a speed of 10,000 rpm for 5 min. Then, the residue was re-dissolved in 4.0 mL of n-hexane. After that, the solution was centrifuged at a rate of 12,000 rpm for 5 min. Finally, the green powders were obtained by vacuum drying. In addition, the effect of LP content on the properties of the prepared samples was investigated, and experiments were carried out as described above.

### 2.3. Synthesis of CsPbBr_3_ Quantum Dots with a Room-Temperature Supersaturated Recrystallization Strategy

As a reference, CsPbBr_3_ quantum dots (QDs) were synthesized using the method reported by Li and coworkers with modifications [32]. A total of 0.8 mmol CsBr, 0.8 mmol PbBr_2_, and 20 mL DMSO were added to a reagent bottle. Then, 2.0 mL OA and 1.0 mL OAm were quickly injected to stabilize the precursor solution. After that, 2.0 mL of the mixed solution were slowly dropped into 20 mL of n-hexane while vigorously stirring. Then, bright-green-emission CsPbBr_3_ QDs were achieved.

### 2.4. Fabrication of Perovskite-based WLED Devices

The perovskite-based WLED devices consist of a green-emitting CsPbBr_3_/Cs_4_PbBr_6_ film, a red phosphor film, and a commercial blue LED chip. The CsPbBr_3_/Cs_4_PbBr_6_ film was fabricated by adding the as-prepared CsPbBr_3_/Cs_4_PbBr_6_ solid powders to PDMS. The mixture was stirred with a vacuum homogenizer for 12 min to degas bubbles. Subsequently, the mixture was injected into a Teflon mold and heated at 120 °C for one hour. The red phosphor film was obtained using the above procedures. Eventually, the WLED devices were fabricated by coating green CsPbBr_3_/Cs_4_PbBr_6_ film and red phosphor film layers on the surface of the blue LED chip.

### 2.5. Measurements of the Visible Light Communication System

The modulations of the bandwidths of the samples were tested by a facile visible light communication (VLC) system [33]. The transmitter of the VLC system mainly includes four parts: an arbitrary waveform generator (AFG, SIGLENT, SDG 6052X-E), a power amplifier (PA, mini circuits, ZHL-6A-S+), a direct-current power supply (DC, Keithley, 2231A), and a bias tee (mini circuit, ZFBT-4R2GW+). A sinusoidal radio frequency (RF) signal is generated by the AFG, which is combined with the DC bias using a bias tee to drive the 450 nm laser diode (LD, YuLiGuangZhou, 450 nm, 15 W). So far, the electric signal has been transformed into a modulated optical signal. Then, divergent and convex lenses were applied to scatter and collate the blue light from the LD. After that, the collimated light excited the green-emitting CsPbBr_3_/Cs_4_PbBr_6_ film and red phosphor film to generate white light. A convex lens is used on the receiver side to gather the modulated white light from the transmitter. An avalanche photodiode (APD, Meno System APD210) was used to convert the optical signal into an electrical signal and amplify it. Finally, the output of the APD was further sampled by an oscilloscope (OSC, SIGLENT, SDS 2000X Plus), which recorded the signals for analysis.

### 2.6. Characterization

The products’ X-ray diffraction (XRD) patterns were determined via an XRD-D8-ADVANCE (Bruker, Bremen, Germany) with a Cu-Kα radiation source. The surface morphology of the samples was characterized by a field-emission scanning electron microscope (FE-SEM, Merlin, Forchtenberg, Germany), and atomic-resolution chemical mapping was achieved using energy-dispersive spectroscopy (EDS) in the FE-SEM. Energy-dispersive X-ray (EDX) spectroscopy (JEOL, Tokyo, Japan) was also carried out to observe element distribution. X-ray photoelectron spectroscopy (XPS) spectra were obtained using a Thermo Scientific (Thermo K-Alpha) (Waltham, MA, USA) machine with a monoatomic Al-Kα excitation source (1486.6 eV). Absorption spectra were performed using a UV-Vis spectrometer (UV-Vis: Tu-1901, Purkinje, Beijing, China). Photoluminescence spectroscopy was implemented by an RF-600 fluorescence spectrophotometer (Shimadzu, Kyoto, Japan), using a xenon lamp as an excitation source. The PLQY measurement of the products was conducted on the Hamamatsu Quantum Yield Measurement System (C9920-02G, Hamamatsu, Japan) under an excitation wavelength of 365 nm. Time-resolved fluorescence spectra were collected by an FLS 980 fluorescence lifetime spectrofluorometer (Edinburgh Instrument, Edinburgh, UK). The PL decay curves obtained are fitted with the multiple exponential functions given in the expression below [30].

(1) $$A(t)=\sum_{i=1}^{n}A_{i}\exp(\frac{-t}{\tau_{i}})$$

where *A*(*t*) represents the PL intensity at time *t*; *A*_i_ denotes the relative weights of the lifetime components at time *t* = 0; and *τ*_i_ represents the decay time for the lifetime components. The average decay lifetime *τ*_avg._ is calculated via the following expression [33]:

(2) $$\tau_{avg.}=\frac{\sum_{i=1}^{n}A_{1}\tau_{i}^{2}}{\sum_{i=1}^{n}A_{1}\tau_{i}}$$

## 3. Results and Discussion

### 3.1. Structural Phase and Morphological Characterizations of As-Prepared Samples

**Figure 2:**
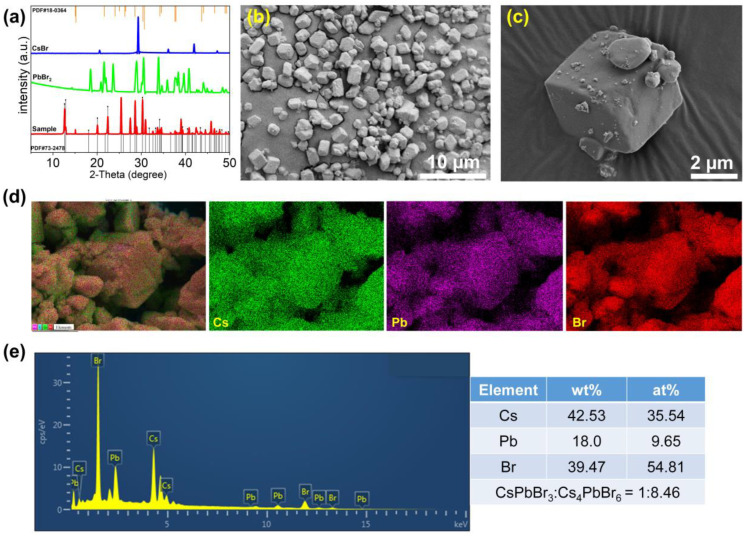
Structure phase and morphological characterization of the as-prepared samples: (**a**) XRD patterns (red lines represent experimental data, and other lines represent standard reference); (**b**) SEM image, scale bar 10 μm; (**c**) high-resolution SEM image (HR-SEM), scale bar 2 μm; (**d**) averaged EDX elemental mapping (green for Cs, purple for Pb, and red for Br) of a small selection of as-obtained samples; (**e**) EDS spectrum and atomic composition.

To identify the crystalline phase of the as-prepared samples, XRD measurements were used. As shown in Figure 2a, the prominent diffraction peaks at 2θ = 12.5°, 12.9°, 20.0°, 22.5°, 25.5°, 28.7°, 30.4°, 31.0°, 34.2°, 38.9°, and 45.7° considerably correspond with the rhombohedral Cs_4_PbBr_6_ (PDF #73-2478) crystal planes (012), (110), (113), (300), (024), (214), (223), (006), (134), (324), and (600) [34]. In addition, we found some characteristic peaks appearing at 2θ = 15.2°, 21.5°, 26.3°, 30.4°, 30.7°, 34.3°, and 43.7°, which can be attributed to the crystal planes (100), (110), (111), (200), (210), (220), and (221) of the cubic CsPbBr_3_ phase (PDF #18-0364) [35]. Furthermore, we compared the as-synthesized samples with standard CsBr and PbBr_2_ XRD spectra and observed whether these phases existed. After careful observation, soft peaks of unreacted PbBr_2_ are observed. Furthermore, no other XRD patterns corresponding to CsBr or other perovskite compounds were detected. Overall, XRD characterization implies that this sample mainly includes CsPbBr_3_ and Cs_4_PbBr_6_ structural phases.

The FE-SEM characterization was performed to observe the morphological features of the samples. As shown in Figure 2b,c, the primary morphology of the sample reaches a micrometer scale. It presents a rhombohedral shape, and its outer surface is embedded with small particles, similar to the previously reported structure of CsPbBr_3_ embedded in Cs_4_PbBr_6_ crystals [30,36]. This result indicates that the sample is CsPbBr_3_/Cs_4_PbBr_6_ composites. In order to quantitatively determine the content of CsPbBr_3_ and Cs_4_PbBr_6_, EDX mapping and EDS characterization were conducted. As shown in Figure 2d, the Cs, Pb, and Br elements are uniformly distributed. Combined with the EDS spectrum, the molar ratio of CsPbBr_3_ and Cs_4_PbBr_6_ is 1:8.46, strongly confirming the co-existence of CsPbBr_3_ and Cs_4_PbBr_6_. To sum up, we successfully prepared CsPbBr_3_/Cs_4_PbBr_6_ composites.

**Figure 3:**
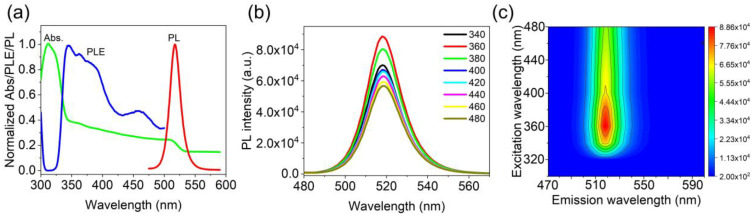
Optical properties of CsPbBr_3_/Cs_4_PbBr_6_ microcrystals: (**a**) normalized photoluminescence (PL) (excited by 365 nm), photoluminescence excitation (PLE) (monitored at 520 nm), and ultraviolet-visible (UV-Vis) absorption spectra; (**b**) PL intensity of the samples under different excitation wavelengths; (**c**) contour plot of the colored PL intensity measured as a function of excitation wavelength.

To investigate the optical properties of the CsPbBr_3_/Cs_4_PbBr_6_ composites, UV-Vis absorption spectroscopy and fluorescence spectroscopy were performed, as shown in Figure 3a–c. The CsPbBr_3_/Cs_4_PbBr_6_ powders demonstrate a strong absorption peak at about 311 nm and a broad absorption band with an absorption edge at 510 nm (Figure 3a). The strong absorption peak located at about 311 nm is agrees well with previous reports on bulk Cs_4_PbBr_6_ powders, further confirming that the isolated octahedral PbBr_6_^4-^ was formed in Cs_4_PbBr_6_ [37]. Another broad absorption at 510 nm differs from previous reports in that the characteristic absorption band of CsPbBr_3_ QD is usually located at 505 nm [38]. This result can explain, to a certain extent, the fact that CsPbBr_3_ has been embedded in Cs_4_PbBr_6_, thereby affecting its absorption. The PLE spectrum of CsPbBr_3_/Cs_4_PbBr_6_ also shows a difference from pure CsPbBr_3_ or Cs_4_PbBr_6_. The fluorescence intensity of the PLE spectrum is relatively low in the short wavelength region (307–325 nm) when the PL emission peak is set at 518 nm and rapidly increases in the 325–345 nm region, extending to longer wavelengths.

Further observation found that the PLE spectrum has a similar change with the absorption spectrum in the range of 345 nm to 505 nm, which is possibly related to the fact that CsPbBr_3_ absorbs the excitation photons and generates fluorescence; thus, a change in the absorption spectrum leads to a corresponding trend in the PLE spectrum. However, in the range of 307 nm to 325 nm, due to CsPbBr_3_ nanocrystals embedded in the Cs_4_PbBr_6_ matrix, the excitation photons are almost entirely absorbed by the Cs_4_PbBr_6_ matrix but not by the internal CsPbBr_3_ nanocrystals. This phenomenon is similar to the pure Cs_4_PbBr_6_ feature [39]. In addition, the PL spectrum of CsPbBr_3_/Cs_4_PbBr_6_ under different excitation wavelengths (340–480 nm) was investigated. As presented in Figure 3b, as the excitation wavelengths increased, the maximum PL emission peak of the CsPbBr_3_/Cs_4_PbBr_6_ showed no changes, indicating high and stable PL emission at 518 nm. An intrinsic emission most likely caused this excitation-independent characteristic. Moreover, as the excitation wavelengths increased, the PL intensity of the sample increased and then decreased, reaching its maximum when the excitation wavelength was 360 nm. This phenomenon can also be observed in the three-dimensional excitation-emission fluorescence spectrum, as shown in Figure 3c. Therefore, combining the PLE spectrum with the three-dimensional excitation-emission fluorescence spectrum, we can conclude that the excitation wavelength of the most substantial emission peak is between 345 nm and 360 nm.

**Figure 4:**
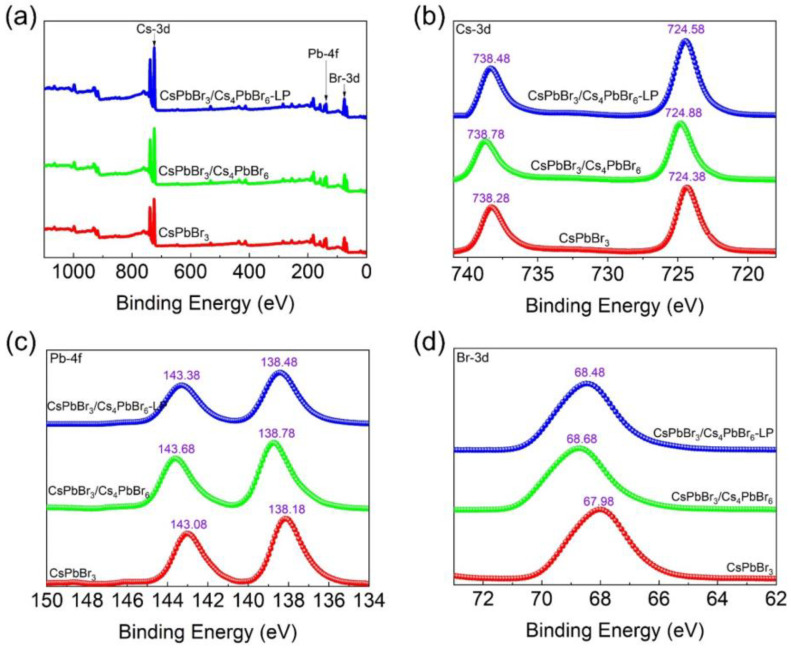
XPS spectra of the CsPbBr_3_/Cs_4_PbBr_6_–LP microcrystals, CsPbBr_3_/Cs_4_PbBr_6_ microcrystals, and pure CsPbBr_3_ QD powders: (**a**) XPS full spectrum; high-resolution XPS analyses corresponding to (**b**) Cs-3d, (**c**) Pb-4f, and (**d**) Br-3d.

XPS was applied to identify the chemical bonding and compositions to further explore the chemical structure of the CsPbBr_3_/Cs_4_PbBr_6_ microcrystals. The CsPbBr_3_/Cs_4_PbBr_6_ and CsPbBr_3_ QDs synthesized without liquid paraffin were used as reference, and CsPbBr_3_/Cs_4_PbBr_6_ synthesized with liquid paraffin was named CsPbBr_3_/Cs_4_PbBr_6_-LP. Figure 4a–d shows the XPS full-scan spectra of the CsPbBr_3_/Cs_4_PbBr_6_–LP, CsPbBr_3_/Cs_4_PbBr_6_, and pure CsPbBr_3_ QD powders and their corresponding high-resolution spectra of Cs-3d, Pb-4f, and Br-3d. Figure 4a demonstrates that three samples are composed of the Cs-3d, Pb-4f, and Br-3d bands. Figure 4b shows that all the Cs-3d spectra possess two peaks with two binding energies, which can be assigned to Cs-3d_5/2_ and Cs-3d_3/2_, respectively. Similarly, the Pb-4f spectra reveal two separated peaks corresponding to the Pb-4f_7/2_ and Pb-4f_5/2_ levels, as shown in Figure 4c. For the Br-3d spectra, all three samples have a broad characteristic peak. After careful comparison, we found that the peaks of Cs-3d, Pb-4f, and Br-3d bands of the CsPbBr_3_/Cs_4_PbBr_6_–LP and CsPbBr_3_/Cs_4_PbBr_6_ shift slightly toward higher binding energies (Figure 4d), which might be attributed to the coating of the Cs_4_PbBr_6_ matrix. Furthermore, compared to the Cs-3d, Pb-4f, and Br-3d bands of the CsPbBr_3_/Cs_4_PbBr_6_, the corresponding peaks in the CsPbBr_3_/Cs_4_PbBr_6_–LP shift to lower binding energies again, suggesting the successful coating of the CsPbBr_3_/Cs_4_PbBr_6_–LP surface with liquid paraffin.

**Figure 5:**
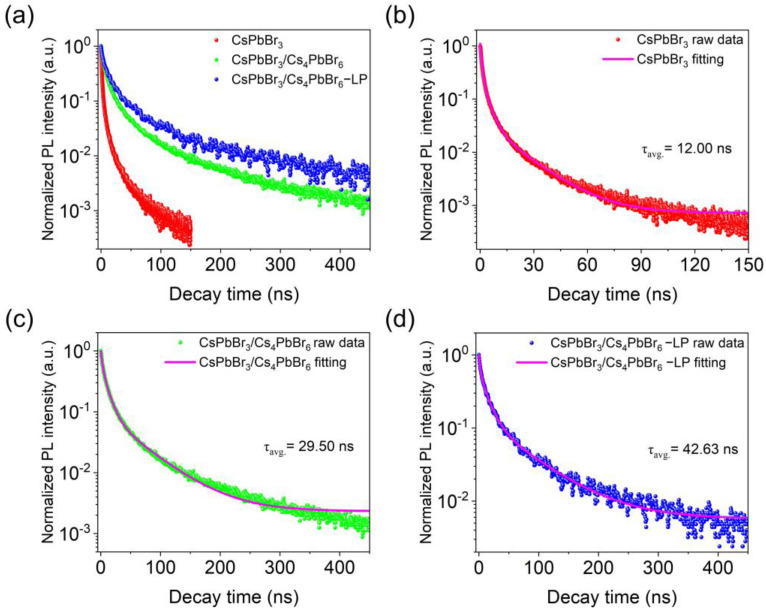
(**a**) The time-resolved PL decay curves of the CsPbBr_3_/Cs_4_PbBr_6_–LP microcrystals, CsPbBr_3_/Cs_4_PbBr_6_ microcrystals, and pure CsPbBr_3_ QD powders. PL decay fitting curves of (**b**) pure CsPbBr_3_ QD powders, (**c**) CsPbBr_3_/Cs_4_PbBr_6_ microcrystals, and (**d**) CsPbBr_3_/Cs_4_PbBr_6_–LP microcrystals.

To analyze PL dynamics for the CsPbBr_3_, CsPbBr_3_/Cs_4_PbBr_6_, and CsPbBr_3_/Cs_4_PbBr_6_–LP, the time-resolved PL decay curves of these three samples were collected using a 375 nm pulse laser as an excitation source. According to Equations (1) and (2), each PL decay curve can be accurately fitted by a triple exponential function, as shown in Figure 5a–d and Table 1. From Figure 5a, the PL lifetimes of the CsPbBr_3_/Cs_4_PbBr_6_ and the CsPbBr_3_/Cs_4_PbBr_6_–LP are relatively prolonged compared with pure CsPbBr_3_. In detail, average PL decay times (τ_avg_.) of 12.00, 29.50, and 42.63 ns for the CsPbBr_3_, CsPbBr_3_/Cs_4_PbBr_6_, and CsPbBr_3_/Cs_4_PbBr_6_–LP, respectively, imply that the Cs_4_PbBr_6_ matrix can passivate the CsPbBr_3_ and liquid paraffin can modify surface defects of CsPbBr_3_/Cs_4_PbBr_6_–LP.

Furthermore, we found that three characteristic time constants τ_1_, τ_2_, and τ_3_ existed in fitting curves, suggesting that the three samples contain more than one emission center with different recombination rates [26]. As previously reported, τ_1_ is attributed to exciton recombination involving surface states and defects, τ_2_ is assigned to radiative recombination, and τ_3_ is related to non-radiative recombination [40]. The amplitude values A_1_, A_2_, and A_3_ are considered the weighing factors [30]. As summarized in Table 1, the time constants τ_1_, τ_2_, and τ_3_ for the CsPbBr_3_ are 1.82, 6.53, and 22.17 ns, respectively. The respective amplitudes A_1_, A_2_, and A_3_ are 0.56, 0.34, and 0.10, respectively. For CsPbBr_3_/Cs_4_PbBr_6_, the time constants τ_1_, τ_2_, and τ_3_ for the CsPbBr_3_ are 2.72, 10.71, and 55.05 ns, and the respective amplitudes A_1_, A_2_, and A_3_ are 0.44, 0.47, and 0.09, respectively. A similar test procedure has been applied to the CsPbBr_3_/Cs_4_PbBr_6_–LP microcrystals. The results for time constants τ_1_, τ_2_, and τ_3_ for the CsPbBr_3_ are 2.27, 12.98, and 67.91 ns, and the respective amplitudes A_1_, A_2_, and A_3_ are 0.38, 0.48, and 0.13, respectively. In terms of corresponding weighing factors A_1_ and A_2_, we found that A_1_ for CsPbBr_3_ is more significant than that of CsPbBr_3_/Cs_4_PbBr_6_ and CsPbBr_3_/Cs_4_PbBr_6_–LP, while A_2_ for CsPbBr_3_ is smaller than that of CsPbBr_3_/Cs_4_PbBr_6_ and CsPbBr_3_/Cs_4_PbBr_6_–LP, indicating that the surface defects of the CsPbBr_3_ are passivated by the well-matched lattice of the Cs_4_PbBr_6_ matrix, thereby increasing the probability of radiative recombination [41]. Furthermore, A_1_ for CsPbBr_3_/Cs_4_PbBr_6_ is more significant than A_1_ for CsPbBr_3_/Cs_4_PbBr_6_–LP, while A_2_ for CsPbBr_3_/Cs_4_PbBr_6_ is smaller than CsPbBr_3_/Cs_4_PbBr_6_–LP, further confirming the passivation of surface defects by liquid paraffin. This result also agrees with the above result that CsPbBr_3_/Cs_4_PbBr_6_–LP possesses a higher PLQY than CsPbBr_3_/Cs_4_PbBr_6_. Therefore, combining Cs_4_PbBr_6_ with liquid paraffin is suitable for better photoluminescence performance.

### 3.2. Effect of the Liquid Paraffin Concentration on the Optical Properties of the CsPbBr_3_/Cs_4_PbBr_6_

**Figure 6:**
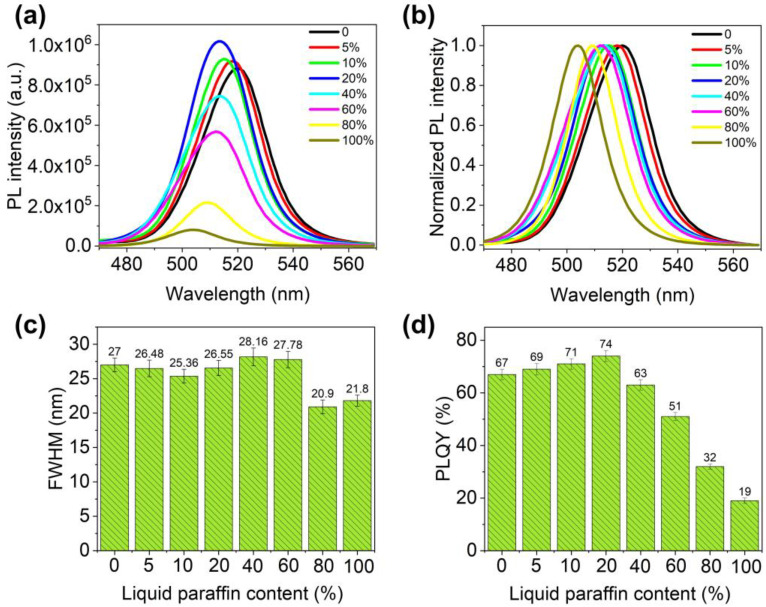
Optical properties of the CsPbBr_3_/Cs_4_PbBr_6_ synthesized by different liquid paraffin contents (0–100%): (**a**) PL emission spectra; (**b**) normalized PL emission spectra; (**c**) FWHM; (**d**) PLQY.

In the preparation process, we found that the contents of liquid paraffin significantly affected the optical properties of CsPbBr_3_/Cs_4_PbBr_6_–LP, so we further investigated this factor. As shown in Figure 6a, as the contents of liquid paraffin increased, the PL intensity of CsPbBr_3_/Cs_4_PbBr_6_–LP increased first. Then, it decreased and was at its maximum when the content of liquid paraffin was 20%, keeping the intensity of the characteristic absorption peak (510 nm) constant. This phenomenon is related to the solubility of CsBr and PbBr_2_ in DMSO and liquid paraffin. Increasing the content of liquid paraffin can improve the protection of samples. However, since CsBr and PbBr_2_ are more difficult to dissolve in liquid paraffin than in DMSO, less CsPbBr_3_/Cs_4_PbBr_6_ is synthesized, and the fluorescence efficiency is lower. In addition, a PL blue shift of the samples, from 518 to 505 nm, was found as the content of liquid paraffin increased (Figure 6b). The reason for this phenomenon is that under different liquid paraffin contents, CsPbBr_3_/Cs_4_PbBr_6_ crystals’ crystallization speed is different, leading to different sizes of CsPbBr_3_/Cs_4_PbBr_6_ composites. When liquid paraffin’s content increases, perovskite’s particle size decreases, and the energy gap widens, so the PL emission wavelength blue-shifts, similar to what has been previously reported [39,42]. Although the content of liquid paraffin is constantly changing, the FWHM of the samples is all less than 30 nm (Figure 6c), which helps to obtain LED devices with high color gamut values. Moreover, the PLQY of the samples varied with the content of liquid paraffin. When the liquid paraffin concentration increased, the PLQY first increased rapidly and then decreased slowly, reaching a maximum of 74% at a liquid paraffin concentration of 20%, which is consistent with the change in PL intensity, as shown in Figure 6d. Therefore, selecting the appropriate liquid paraffin concentration is crucial to obtaining high optical performance.

### 3.3. Stability of CsPbBr_3_/Cs_4_PbBr_6_ Microcrystal

**Figure 7:**
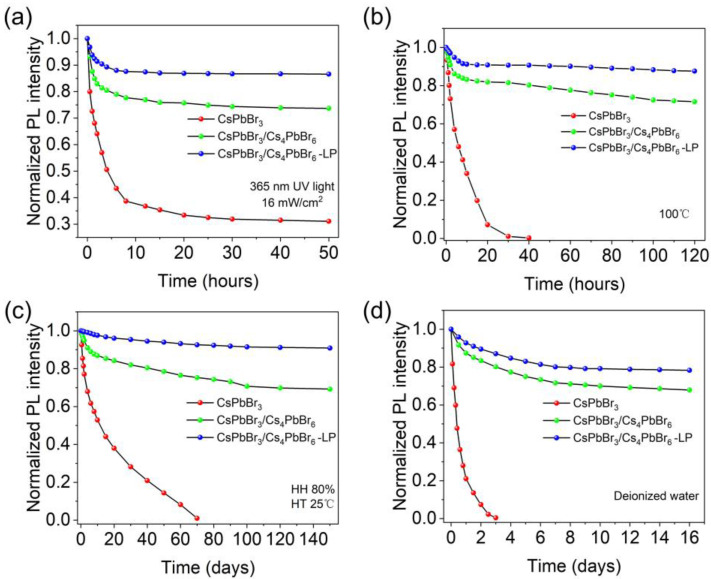
UV photostability, thermotolerance, storage stability, and polar solvent stability of pure CsPbBr_3_ QD powders, CsPbBr_3_/Cs_4_PbBr_6_ microcrystals, and CsPbBr_3_/Cs_4_PbBr_6_–LP microcrystals: (**a**) PL intensity of three samples under continuous illumination by 365 nm UV light, power density 16 mW/cm^2^; (**b**) Time-dependent PL intensity stability after heating at 100 °C for various times ranging from 0 to 120 h; (**c**) PL intensity of three samples after 150 days of storage under ambient conditions (HH 80% and HT 25 °C); (**d**) PL intensity of three samples soaked in deionized water (30 mg/mL) after 16 days.

The performance of perovskite crystals is severely affected by the surrounding environment, such as heat, ultraviolet light, and polar solvents. The stability of perovskite crystals is crucial for their practical applications. Here, we systematically investigated the UV photostability, thermotolerance, storage stability, and water stability of CsPbBr_3_/Cs_4_PbBr_6_ powders. To evaluate the UV light resistance of the as-obtained samples, CsPbBr_3_, CsPbBr_3_/Cs_4_PbBr_6_, and CsPbBr_3_/Cs_4_PbBr_6_–LP powders were irradiated under continuous 365 nm UV light with an optical power density of 16 mW/cm^2^ for 50 h, as shown in Figure 7a. The normalized PL intensity of CsPbBr_3_/Cs_4_PbBr_6_–LP presented only a 13.4% decrease after the irradiation of 50 h, while CsPbBr_3_ and CsPbBr_3_/Cs_4_PbBr_6_ dropped 68.9% and 26.3% at the same measurement conditions, respectively. This excellent photostability of CsPbBr_3_/Cs_4_PbBr_6_–LP is mainly due to the double protection of liquid paraffin and the Cs_4_PbBr_6_ matrix. In addition, the thermotolerance of CsPbBr_3_, CsPbBr_3_/Cs_4_PbBr_6_, and CsPbBr_3_/Cs_4_PbBr_6_–LP was investigated. Figure 7b shows the normalized PL intensity change of three sample powders heated at 100 °C within 120 h. The results show that the CsPbBr_3_/Cs_4_PbBr_6_–LP decays only 12.5%, while the CsPbBr_3_ is nearly fluorescence-quenched, and the CsPbBr_3_/Cs_4_PbBr_6_ drops 38.4% after heating for 120 h, which further verifies the satisfactory thermotolerance of CsPbBr_3_/Cs_4_PbBr_6_–LP.

Water and air resistance are also essential for perovskite materials. We further study the storage and polar solvent stability of CsPbBr_3_, CsPbBr_3_/Cs_4_PbBr_6_, and CsPbBr_3_/Cs_4_PbBr_6_–LP. As shown in Figure 7c, the PL intensity of CsPbBr_3_/Cs_4_PbBr_6_–LP dropped only 9.1% after 120 days of storage under ambient conditions (HH 80% and HT 25 °C), while CsPbBr_3_ and CsPbBr_3_/Cs_4_PbBr_6_ attenuated 99% and 30.8%, suggesting that our CsPbBr_3_/Cs_4_PbBr_6_–LP possesses stable green emission ability. Additionally, the polar solvent stability of three samples was evaluated, as shown in Figure 7d. Surprisingly, the CsPbBr_3_/Cs_4_PbBr_6_–LP keeps 78% of its initial PL intensity after being soaked in water (30 mg/mL) for 16 days, while CsPbBr_3_ decay is swift, and it eventually loses its luminescence. The CsPbBr_3_/Cs_4_PbBr_6_ maintains 67% of its initial PL intensity. Therefore, these results demonstrate that our CsPbBr_3_/Cs_4_PbBr_6_–LP possesses excellent photostability, thermal stability, storage stability, and polar solvent stability, showing great potential as high-quality optoelectronic devices under harsh conditions.

### 3.4. Application in WLEDs and Visible Light Communication Devices

**Figure 8:**
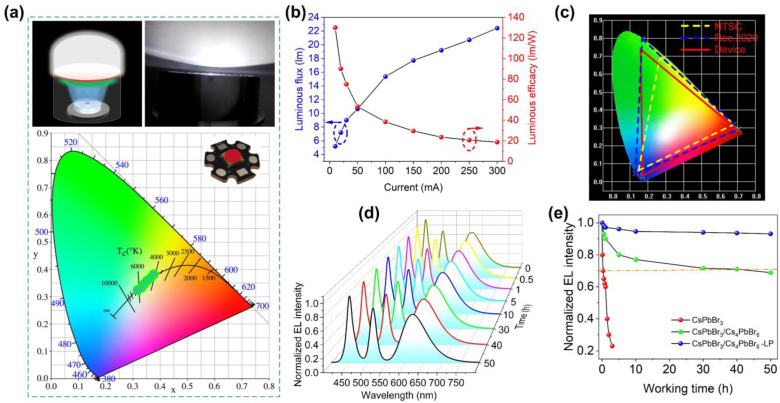
(**a**) A schematic and physical diagram of CsPbBr_3_/Cs_4_PbBr_6_–LP WLED devices and their corresponding color coordinates in a CIE diagram at different forward-bias currents (10–300 mA). (**b**) The changes in luminous efficiency and luminous flux of the CsPbBr_3_/Cs_4_PbBr_6_–LP WLEDs at different forward-bias currents. (**c**) Color coordinates and color gamut of CsPbBr_3_/Cs_4_PbBr_6_–LP WLEDs plotted on the CIE1931 chromaticity diagram. CsPbBr_3_/Cs_4_PbBr_6_–LP WLED device (red line), NTSC standard (yellow dashed line), and Rec. 2020 standard (blue dashed line). (**d**) The electroluminescence (EL) spectra of the WLEDs were measured at different working times (0–50 h). (**e**) Comparison of the normalized green light EL intensity of the WLED devices using pure CsPbBr_3_ QD powders, CsPbBr_3_/Cs_4_PbBr_6_ microcrystals, and CsPbBr_3_/Cs_4_PbBr_6_–LP microcrystals at different working times.

Benefiting from the good optical properties and excellent stability of CsPbBr_3_/Cs_4_PbBr_6_–LP, its application in display and visible light communication devices is promising. Firstly, we fabricated a WLED device with a commercial blue LED chip, green-emissive CsPbBr_3_/Cs_4_PbBr_6_–LP film, and red phosphor film; the schematic and physical diagram of WLEDs are shown in Figure 8a. When the CsPbBr_3_/Cs_4_PbBr_6_–LP powder concentration was adjusted from 1.5 wt% to 10 wt%, the correlated color temperature (CCT) of the WLED devices ranged from 4100 K to 6500 K, and their color rendering index (CRI) was more than 85. In addition, with increased CsPbBr_3_/Cs_4_PbBr_6_–LP powder concentration, the CCT value changes along the Planckian locus line, demonstrating a promising candidate for display. The driving current-dependent luminous flux and efficacy of the CsPbBr_3_/Cs_4_PbBr_6_–LP WLED device are presented in Figure 8b. The luminous efficacy curve shows that the luminous efficacy of the remote excitation LED device reaches 129.5 lm/W before aging, which is comparable to the highest efficiency record of the traditional ligand-protected CsPbBr_3_ QDs. Compared with the expected standard, the CsPbBr_3_/Cs_4_PbBr_6_–LP-based WLEDs have a wide color gamut (solid red triangle), with 121% of the NTSC (white dashed line) and 94% of the Rec. 2020 (blue dashed line), as demonstrated in Figure 8c.

In addition to analyzing the CsPbBr_3_/Cs_4_PbBr_6_–LP-based WLED device’s optical performance, we also evaluated its reliability. The LED industry usually adopts the double 85 standards. The product is aged in an environment with a high temperature of 85 °C and a high humidity of 85% (85 °C/85% RH). The performance changes before and after aging are compared to determine the product’s heat and humidity resistance. Here, we put the WLED device in an environment of 85 °C for thermal reliability experiments. The changes in optical performance during the aging process are shown in Figure 8d. After aging for 50 h, the EL spectrum shape remains almost unchanged, and the intensity of the green light spectrum decreases slowly. It can be seen from the normalized data that although the green light decreases after aging (Figure 8e), the aging time for its spectral intensity to decay to 90% of the initial intensity is as long as 50 h and gradually tends to be stable. In contrast, under the same conditions, WLED devices using CsPbBr_3_ decayed to about 70% of their initial intensity in less than an hour. In comparison, WLED devices containing CsPbBr_3_/Cs_4_PbBr_6_ decayed to 68% of their initial intensity in 50 h, which shows that CsPbBr_3_/Cs_4_PbBr_6_–LP-based WLED devices have good thermal reliability.

**Figure 9:**
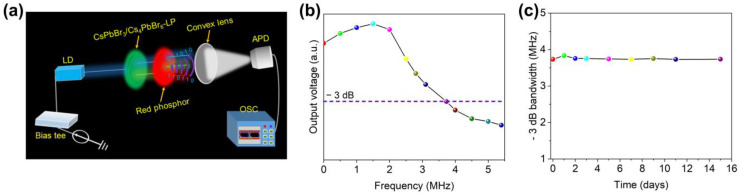
(**a**) Schematic diagrams of the VLC test system. (**b**) Frequency response of the CsPbBr_3_/Cs_4_PbBr_6_–LP-based WLEDs. (**c**) The −3 dB bandwidth of CsPbBr_3_/Cs_4_PbBr_6_–LP-based WLEDs after exposure to the air.

Besides the display, CsPbBr_3_/Cs_4_PbBr_6_–LP-based WLEDs can also be used as optical sources to transmit data in VLC systems. Here, we investigated the communication performance of CsPbBr_3_/Cs_4_PbBr_6_–LP-based WLEDs using the measurement setup shown in Figure 9a. The electrical–optical–electrical frequency response of the device can be obtained using such a test system. CsPbBr_3_/Cs_4_PbBr_6_–LP-based WLEDs were tested at a direct current bias of 3.0 V. From Figure 9b, it can be seen that these devices exhibit a typical low-pass frequency response, corresponding to a −3 dB bandwidth of about 3.7 MHz. Compared with the conventional phosphor white light system, the PL lifetime of CsPbBr_3_/Cs_4_PbBr_6_–LP (nanoseconds) is much shorter than phosphor (microseconds). According to previous reports, the −3 dB bandwidth of the CsPbBr_3_/Cs_4_PbBr_6_–LP-based WLEDs also can be calculated by Equation (3) [7,43]:

(3) $$F_{\left(-3dB\right)}=\frac{1}{2\pi \tau_{avg.}}$$

Thus, the bandwidth estimation of the CsPbBr_3_/Cs_4_PbBr_6_–LP-based WLEDs is 3.733 MHz, consistent with the result collected from Figure 9b. In addition, the time-dependent −3 dB bandwidth of the CsPbBr_3_/Cs_4_PbBr_6_–LP-based WLEDs was measured after exposure to the natural environment. Figure 9c exhibits that the CsPbBr_3_/Cs_4_PbBr_6_–LP-based WLED device shows almost no decay after 15 days in the air (20–28 °C), suggesting that it has good stability and is promising in communication applications.

## 4. Conclusions

In summary, we demonstrated a facile and effective strategy to enhance the performance of CsPbBr_3_/Cs_4_PbBr_6_, which was performed by applying ultrasonication and liquid paraffin. By applying XRD, SEM, EDX, EDS, Abs/PL/PLE, XPS, and PL decay lifetime characterizations, all these results provide solid evidence supporting the formation of CsPbBr_3_/Cs_4_PbBr_6_ composites. Changing the content of liquid paraffin, bright-emission CsPbBr_3_/Cs_4_PbBr_6_-LP solid powders with a maximum PLQY of 74% and a narrow FWHM of about 27 nm were achieved. Thanks to the protection of the Cs_4_PbBr_6_ matrix and liquid paraffin, the PL intensity of CsPbBr_3_/Cs_4_PbBr_6_-LP dropped only 13.4% after continued irradiation by 365 nm UV light for 50 h and decayed only 12.5% at 100 °C within 120 h. Moreover, the CsPbBr_3_/Cs_4_PbBr_6_-LP powder shows superior stability with minimal degradation after 120 days of storage under ambient conditions. Even after soaking in a polar solvent (water) for 16 days, its PL intensity remained at about 85% of the initial value. The fabricated CsPbBr_3_/Cs_4_PbBr_6_-LP-based WLEDs show excellent luminescent performance, with a power efficiency of 129.5 lm/W and a wide color gamut, with 121% of the NTSC and 94% of the Rec. 2020, suggesting they represent a promising candidate for displays. In addition, the CsPbBr_3_/Cs_4_PbBr_6_-LP-based WLEDs were also demonstrated in a VLC system. The results suggested the great potential of these high-performance WLEDs as an excitation light source to achieve visible light communication.

## Figures and Tables

**Table 1:** Fitted lifetimes of the CsPbBr_3_, CsPbBr_3_/Cs_4_PbBr_6_, and CsPbBr_3_/Cs_4_PbBr_6_–LP.

Samples	A_1_	τ_1_ (ns)	A_2_	τ_2_ (ns)	A_3_	τ_3_(ns)	τ_avg._ (ns)
CsPbBr_3_/Cs_4_PbBr_6_-LP	0.38	2.27	0.48	12.98	0.13	67.91	42.63
CsPbBr_3_/Cs_4_PbBr_6_	0.44	2.72	0.47	10.71	0.09	55.05	29.50
CsPbBr_3_	0.56	1.82	0.34	6.53	0.10	22.17	12.00

## Data Availability

The data presented in this study are available on request from the corresponding author.
